# Neuroendocrine regulation of female aggression

**DOI:** 10.3389/fendo.2022.957114

**Published:** 2022-08-10

**Authors:** Vinícius Elias de Moura Oliveira, Julie Bakker

**Affiliations:** Laboratory of Neuroendocrinology, GIGA-Neurosciences, University of Liege, Liege, Belgium

**Keywords:** aggressive behavior, social behavior, estrogen receptors, oxytocin (OXT), vasopressin (AVP), corticosterone, sex differences, aromatase

## Abstract

Classically the neurobiology of aggression has been studied exclusively in males. Thus, females have been considered mildly aggressive except during lactation. Interestingly, recent studies in rodents and humans have revealed that non-lactating females can show exacerbated and pathological aggression similarly to males. This review provides an overview of recent findings on the neuroendocrine mechanisms regulating aggressive behavior in females. In particular, the focus will be on novel rodent models of exaggerated aggression established in non-lactating females. Among the neuromodulatory systems influencing female aggression, special attention has been given to sex-steroids and sex-steroid-sensitive neuronal populations (i.e., the core nuclei of the neural pathway of aggression) as well as to the neuropeptides oxytocin and vasopressin which are major players in the regulation of social behaviors.

## Introduction

Typically aggressive behavior is defined as an innate social behavior expressed whenever animals face conflicts over essential resources for their survival such as mates, food, water, and/or territory (1, 2). The fact that in nature aggression is displayed in those territorial settings has led scientists to assume that females are only, if so, mildly aggressive (1–4) except during the physiologically unique period of lactation where females will exacerbate their aggressive behavior to defend their offspring (5). These assumptions come from the fact that males and females differ significantly in terms of body size and weaponry to aggress (claws, canines, and horns) as well as in selective pressure, regarding their reproductive fitness (4, 6, 7). In general, males tend to adopt a polygynous strategy to guarantee their reproductive success, meaning that males will try to mate with as many females as possible. To accomplish that, they will need to gain access to more resources (food, water, and/or territory) to find their mates and to have the physical strength necessary to protect those mates against possible competitors (1, 8).

By contrast, according to this theory, female reproductive fitness is not based on a polygynous strategy as females are generally fertilized by one male (in most vertebrate species at least) (9). Thus, in theory, females do not need to compete for those resources. In addition, aggression might be a risk for females as the possibility of severe injury could strongly decrease their reproductive success (8). However, once pregnancy and lactation are achieved, this picture shifts as females invest a lot of their energy in pregnancy, lactation, and maternal care. Therefore, exacerbated maternal aggression becomes an adaptative response to protect their offspring and consequently achieve reproductive success (4–6, 8).

Although this evolutionary theory of sexual dimorphism on aggression appears quite appealing at first sight, it is necessary to point out that females might also compete for crucial resources for their survival especially when those resources are limited. For example, females need to get access to nutrition and territory as those are necessary for fitness. Thus, one could hypothesize that females compete to gain access to the best options available (7, 10). When it comes to sexual courtship and mate choice, females for may also compete for the “fittest” mates, with perhaps the best examples the lekking topi antelopes and female prairie voles which actively compete for sexual partners (10). Female voles develop wander behavior with polyandrous (mating with several males) strategy similarly to wander males (polygynous) (11), which is also accompanied by increased aggression and decreased affiliative behaviors towards other females (12). Additionally, one could predict that females need to be able to reject unsuitable mating partners and defend themselves in case of retaliation. Altogether, those scenarios illustrate some situations, in which non-lactating females should display some levels of aggression in order to either protect themselves from threats or to guarantee their survival and/or fitness.

Accordingly, aggression in territorial/”rivalry” related conditions have been reported in females of several species from invertebrates, such as fruit flies (13, 14) and octopus (15) to vertebrates, such as fish (7), birds (16, 17), rodents (mice (18–22), rats (23–28), hamsters (29–35), mole rats (6), voles (12), lemurs, marmoset, hyenas (6), antelopes (10), and humans (8, 36–38). Despite this compelling evidence of the occurrence of female aggression in nature, the neural substrates and neuroendocrine factors underlying this behavior have only recently gained scientific attention. In fact, the neurobiological underpinnings of non-maternal female aggression are still relatively unknown in comparison to intermale aggression.

Here it is important to highlight that besides showing adaptative aggression, females might also develop pathological and disruptive aggression. For instance, recent reports in humans have demonstrated that girls and women develop aggression disorders such as conduct and antisocial personality disorder similarly to boys and men, respectively (8, 36–39). Thus, the study of the neurobiological mechanisms underlying female aggression is not only relevant for basic but also for translational neuroscience research in order to find suitable treatment options for aggression disorders in both sexes.

In this review, we will describe the recent discoveries on the neuroendocrine mechanisms regulating female aggression especially focusing on non-lactating animal models of female aggression. Additionally, we will particularly emphasize the role of neurosteroid-responsive neurons, core regulators of the neural pathway of aggression (1), as well as the neurohypophyseal hormones oxytocin and vasopressin, major modulators of social and aggressive behaviors (40).

## Maternal aggression

In most rodent species, females will typically exhibit a dramatic increase in their aggressive behavior during the first week of lactation, the so-called maternal aggression. Maternal aggression wanes over the last half of lactation, disappearing around weaning or when pups are culled (3, 5, 18). Conventionally, maternal aggression is considered an important part of the repertory of behaviors displayed by lactating rodents as the survival of pups depends on a combination of maternal care and maternal aggression (5). In fact, several authors do not consider maternal aggression as a typical antisocial behavior, but rather a prosocial type of aggression as it is displayed not to protect oneself, but rather to protect others (5, 41, 42). Despite this hypothesis, maternal aggression is a highly adaptative behavior that assures fitness similarly to territorial aggression (1, 4, 5).

Interestingly, although the pure levels of maternal aggression do not seem to differ from the levels of aggression displayed by males in the resident intruder test (RIT) (18, 43), qualitatively, lactating females seem to show severer attacks than males, as they attack vulnerable targets such as the head and genitals (43). From a neurobiological point of view, various neuropeptitidergic, as well as hormonal systems, are known to be up-regulated during lactation and involved in the expression of maternal aggression (4, 5). Of note, it is important to mention the participation of oxytocin (OXT) (5), vasopressin (AVP) (5), corticotropin-releasing hormone (CRH) (44), gonadotropin-releasing hormone (GnRH) (45), prolactin (46), estrogen receptor α (Erα)- (47), and aromatase-expressing neurons (43).

Importantly, when pertinent, we will make a parallel between the systems involved in maternal and non-maternal female aggression.

## Rodent models of non-maternal female aggression

### Naturally aggressive species

Although females are assumed to be less aggressive than males, in some rodent species, virgin females are known to display high levels of aggression, for a review please see (6, 35). Among those species, we will particularly focus on hamsters and California mice due to the important behavioral neuroendocrinology research conducted over the last decades on those animals.

### Hamsters

Female Syrian hamsters (*Mesocricetus auratus*) are highly territorial and show aggression towards male and female intruders. In fact, female hamsters will only reduce their aggression towards males when they are sexually receptive (32–35, 48). Additionally, external factors such as social isolation (48) and successive aggressive interactions (32, 34) escalate the already high levels of aggression displayed by those animals.

### California mice

Virgin female *Peromyscus californicus* are known to display high levels of aggression towards female intruders. Aggression was found to be especially dependent on photoperiod and experience, with higher scores on short days and after multiple testing (19). Notably, aggression in this species seems to be independent of the estrous cycle (22).

### Animal models of “territorial” aggression in a resident-intruder setting (mice and rats)

#### Confrontation with a juvenile

Although anecdotal data has described aggression among wild female mice, most of the studies using laboratory mouse strains have reported mild or no aggression in female mice (3, 4). However, recent findings have shown striking strain differences in naturally occurring aggression in female mice. Swiss but not C57BL/6N females display aggression towards male juveniles (in 80% of the encounters) and adult female intruders (in about 60% of the encounters). In those animals, social isolation was also reported to enhance aggression. Additionally, the estrous cycle of the resident female was found to not influence aggressive behavior (47).

#### Co-housing

Co-habitating a male resident with a stimulus female is a well-established method to induce reliable levels of aggression in male mice (3) and rats (49, 50). However, until recently this protocol was never used in females. Newman et al. (18) have shown that cohabitation with a male significantly increases the levels of aggression exhibited by Swiss female mice, which could be reduced by either social isolation or ovariectomy. Additionally, after pup culling, postpartum mice recovered their levels of aggression after being cohoused with gonadectomized males. Accordingly, nulliparous females cohoused with gonadectomized males displayed high levels of aggression (65% of the females presented more than 15 bites within the first 2 minutes of interaction), which were similar to the levels normally displayed by male mice, with females showing more rapid bite bouts and pursuit bites than males. Also in this model, no apparent effect of the estrous cycle on female aggression was found (18).

Recently, a new study has refined these findings by showing that co-housed CFW mice display similar levels of aggression independent of sex. Interestingly, although mice did not differ in attack latency and duration, they exhibited striking differences in the composition of their aggressive behavior with males displaying more wrestling and lung (threat-like behavior) compared to females. Females on the other hand showed a higher number of attack bites. Sex differences also emerged when the Hidden Markov Model was applied to characterize the behavioral strategies used by both sexes. Briefly, males engaged in persistent attack bouts (characterized by the occurrence of only aggressive behavior) whereas females displayed intermittent attacks which occurred in association with investigative social behaviors. This suggests that males show a more marked transition between investigative social interactions and aggressive behavior, whereas females appear to display mixed interactions (20). Furthermore, together with the fact that females display less wrestling and lung behavior, this indicates that aggression in female mice has a reactive and/or defensive phenotype, suggesting that they do not seek aggressive interactions. In fact, in the same study, aggressive interactions were not rewarding and/or reinforcing (20) in females in the aggression-conditioned place preference test (51) and aggression operant self-administration procedure (52).

Similar results have been reported in an early study in female Wistar rats (24) where females were co-housed with a sterilized (ligation and incision of the vasa deferentia) male to induce pseudopregnancy. After pseudopregnancy vanished, those animals were found to display aggression towards an ovariectomized intruder. Differently from mice, in rats, aggression was found to be dependent on the estrous cycle as females in estrus were less aggressive than females in metestrus and diestrus (24).

In female prairie voles, cohabitation with a male is also known to enhance aggression and diminish affiliative behaviors (12). Likewise, cohabitation and the development of a pair bond have been suggested to increase aggression towards same-sex intruders in female California mice (21, 22).

#### Social isolation and aggression-training

Wistar rats typically display mild levels of aggression, regardless of sex (24, 28, 49, 50, 53). Nevertheless, male Wistar rats have been consistently used to study aggression in combination with the most diverse paradigms to induce high levels of aggression, for a review please see (49, 54, 55). Females, on the other hand, have been poorly studied. Recent findings using the female intruder test (FIT), a direct analog to the RIT, where an intact normally cycling female is confronted with a smaller (10-20% lighter) same-sex intruder for 10 min, showed that females display “rivalry” aggression towards conspecifics (25). Interestingly, group-housed male and female Wistar rats did not differ significantly in their levels of aggression (25, 28). Additionally, neither intruder nor the resident’s estrous cycle had an apparent effect on aggression (25).

Using the same paradigm, Oliveira et al. (23) found that a combination of social isolation (9 days) accompanied by successive aggressive interactions, i.e. daily 10 min exposition to a FIT in three consecutive days, strongly exacerbated the mild levels of aggression displayed by virgin female Wistar rats. Both socially isolated (IS) and isolated and trained (IST) females displayed higher levels of aggression (>20% on average) than non-trained group-housed (GH) females (<10% on average). Interestingly, isolation-induced aggression was affected by the estrous cycle as no increase in aggression was found in IS females in proestrus or estrus. The effects of the estrus cycle completely vanished in IST females (23).

### Animal models of abnormal and/or pathological aggression

Behavioral neurobiologists have established rodent models of excessive and pathological aggression using male Wistar rats as model organisms to understand which are the mechanisms underlying disruptive aggression in humans. Those models are known to exhibit the so-called abnormal aggression which is characterized by: i) mismatch between provocation and response (increased number of attacks or attack in neutral arenas); ii) disregard for species-specific rules (attacking juveniles, females, or vulnerable targets, such as gonads, paws, head or belly) and iii) insensitivity towards social signs of the intruder (attacking anesthetized and/or submissive intruders), for a review please see (55, 56).

#### Rats selectively bred for low and high anxiety-related behavior

Male rats selectively bred for low (LAB) and high (HAB) anxiety-related behavior display high and abnormal levels of aggression (57, 58). Particularly, LABs are known to be extremely aggressive towards females, anesthetized intruders, and to attack vulnerable targets (57). By contrast, maternal (5) and non-maternal (25) female aggression were found to be higher in HAB rather than in LAB rats, implying a sex-specific effect of the trait of anxiety on aggressive behavior. In fact, in virgin females, time spend in the open arm of the elevated plus-maze negatively correlated with aggression, meaning that a highly anxious female also showed high levels of aggression (25). Yet, it remains to be assessed whether HAB and LAB females show abnormal aggression similarly to males.

#### Peripubertal stress

Briefly, exposition to a combination of fear-inducing stressors such as an open field, an elevated platform in bright-light conditions, and the synthetic fox odor trimethylthizoline (TMT), is shown to induce excessive and abnormal aggression in male Wistar rats (i.e., attack to vulnerable targets, smaller and anesthetized intruders) (56, 59). Importantly, in this protocol, animals are exposed (for 25 min) to those stressors in unpredictable and semi-random order with one or two stressors a day, during the peripubertal period, from postnatal day 28 until 42 (p28 to p42, a total of 7 days) (56, 59).

In females, peripubertal stress was found to increase aggression towards an ovariectomized intruder in the RIT, independently of the phase of the estrous cycle. Those animals were also reported to exhibit aggression towards males during the cohabitation period prior to parturition and to develop higher levels of maternal aggression (shorter attack latency), compared to non-stressed controls (26). It remains to be verified whether PB also induces other signs of abnormal aggression in females (such as attacks towards vulnerable targets or juveniles). Nevertheless, this model confirms the deleterious effects of stress during early life on aggressive behavior, independent of sex.

#### Post-weaning social isolation

Social deprivation after weaning (p21) was described to robustly enhance the levels of aggression displayed by male Wistar rats and mice (56, 60, 61). Specifically, in male rats, PWSI is known to induce exaggerated as well as abnormal aggression (consisting of hard bites, attacks towards vulnerable targets, and non-signaled attacks, i.e. without threat or from defense) (61).

Similar results have been found in females as PWSI induced excessive and abnormal aggressive behavior in virgin female Wistar rats. Although isolated males and females did not differ in total levels of aggression, the number of attacks, and attacks towards vulnerable body parts, abnormal aggression was displayed in a sex-specific manner, with females showing higher levels of aggression towards juveniles and males tending to show a higher number of attacks and attacks towards vulnerable targets (28). Similar to PB, these data depict PWSI as a robust and reliable model to induce excessive and pathological aggression in both sexes.

## Neuroanatomy of female aggression

Although the neurocircuitry of maternal aggression has been extensively characterized over the last decades (62, 63), the neural substrates of aggression in virgin/non-lactating females are largely unknown. A couple of studies using either the expression of immediate early genes and/or longitudinal *in vivo* calcium recordings (fiber photometry) have revealed some brain regions which might be implicated in the display of aggression by non-lactating females, for an overview please see **Table 1**.

**Table 1:** Overview of studies assessing neural activity during/after virgin female aggression.

Brain region	Species	Activity	Marker	Reference
*Hypothalamus*				
HAA	Rat	↑	pERK	(25)
PVN	Rat	↓	pERK	(25)
PVN (OXT)	Rat	↓	pERK	(25)
PVN (AVP)	Hamster	↔	Fos	(30)
VMHvl (Erα/Npyr2)	Mice	↑	*In vivo* calcium recordings/Fos	(47, 64)
*Limbic regions*				
BNST	California mice	↑	pERK	(19)
CeA	Rat	↑	pERK	(27)
MeA	California mice	↑	pERK	(19)
dLS	Rat	↓	pERK	(23)
vLS	Rat	↑	pERK	(23)
*Brainstem*				
PAG	Mice	↑	Fos	(47)
Dorsal Raphe (5-HT)	Hamster	↑	Fos	(30)

The table highlights the species and neural activity marker/method used. HAA, Hypothalamic attack-area; PVN, paraventricular nucleus of the hypothalamus; OXT, oxytocin; AVP, vasopressin; VMHvl, ventromedial hypothalamus ventrolateral part; Erα, Estrogen receptor alpha; Npyr2, Neuropeptide Y receptor 2; BNST, bed nucleus of stria terminalis; CeA, Central amygdala; MeA, medial amygdala; dLS, dorsal lateral septum; vLS, ventral lateral septum; PAG, periaqueductal gray matter; 5-HT, serotonin; ↑, increased activity; ↓, decreased activity; ↔ unchanged.

Concerning hypothalamic areas, aggression was shown to activate the medial portion of the ventrolateral part of the ventromedial nucleus of the hypothalamus (VMHvl) (47, 64) as well as the hypothalamic attack area [HAA, mediobasal hypothalamus (55)] (25) in virgin female mice and rats, respectively. Additionally, in female Wistar rats, aggression was found to reduce the number of phosphorylated ERK (pERK) positive cells in the paraventricular nucleus of the hypothalamus (PVN) (25). Regarding limbic regions, the bed nucleus of stria terminalis (BNST) and the medial amygdala (MeA) show increased pERK after aggression (19) in California mice. In highly aggressive IST female rats, GABAergic neurons within the ventral lateral septum (vLS) (23), as well as the central amygdala (CeA) (27), were found to be activated after FIT exposure. By contrast, in the dorsal lateral septum (dLS), GABAergic neurons showed reduced pERK after the FIT. Additionally, activity (pERK/VGAT colocalization) negatively correlated with aggression in those animals (23). The participation of those subregions of the LS on female aggression was further confirmed using pharmacology: muscimol infusion into the dLS enhanced whereas, in the vLS, it decreased female aggression in Wistar rats (23). Regarding the brainstem, aggression was found to activate the lateral periaqueductal gray matter in female mice (47). Although those studies indicate the participation of crucial hubs regulating intermale aggression such as the LS (65) and mediobasal hypothalamus (66) also in female aggression, future studies should focus on dissecting those pathways and better characterizing the connections among those regions.

## Neuroendocrinology of female aggression

### The role of sex-steroid and sex-steroid-responsive neurons

Sex steroids (estradiol, progesterone, and testosterone) are mainly produced in the gonads under the influence of the hypothalamus-pituitary-gonad (HPG) axis. Briefly, gonadotropin-releasing hormone (GnRH) will be released from the hypothalamus into the median eminence acting in gonadotropes at the anterior pituitary and triggering the release of luteinizing (LH) and follicle-stimulating hormones (FSH), which will then act at the gonads stimulating the production and release of sex-steroids (67, 68). In turn, sex steroids will travel back to the brain and act on neurons generating a negative feedback loop (68) but also modulating behavior (69–71).

Social behaviors such as social investigation, sexual and aggressive behavior, are influenced by sex steroids (69–71). Particularly estrogen receptor alpha (Erα) is expressed in the main nuclei embedded in the neural pathway of aggression such as the MeA, the lateral septum (LS), the BNST, the medial pre-optic area (MPOA), and VMHvl (1, 4, 72, 73). This pattern of expression together with the fact that this receptor exerts a crucial role in intermale aggression (1, 4, 71), has raised the hypothesis that sex differences in aggressive behavior are mainly driven by masculinization of the neural network of aggression by aromatized estradiol (4). Accordingly, aromatase knockout (ArKO) males show feminized brain responses (74) and reduced aggressive behavior towards a male but increased aggressive behavior towards an estrous female (75). Additionally, masculinization of the female brain, i.e. by exposing females to testosterone or estrogen during perinatal development, has been shown to exacerbate aggression in females as well as to make females more sensitive to the pro-aggressive activational effects of testosterone in adulthood, by increasing female attacks towards male intruders (76).

Although the data described above suggest an interaction between the organizational (70) and activational (70) effects of estrogens on the neural pathway of aggression to orchestrate the sex-specific behavioral patterns seen in adults, more research is needed. It is unlikely that femininization of the brain hubs controlling aggression completely abolishes aggressive behavior in females since females exhibit extreme levels of maternal aggression during lactation (5, 18). Thus, one could hypothesize that sex hormones would rather modulate or temporarily suppress female aggression.

Conversely, female aggression in several rodent species was found to be independent of the estrous cycle (18, 19, 22, 23, 25, 26, 28, 47), indicating that subtle alterations in plasma concentrations of sex steroids are unlikely to modulate aggression in females. Nevertheless, a minimum concentration of circulating sex hormones seems to be necessary for the display of aggression in females as ovariectomy completely suppressed aggression in female mice and rats, respectively (18, 24). Also, hormonal alterations and treatments were associated with aggression in female rodents. For instance, a reduced progesterone/testosterone ratio was found in the plasma of California mice after a confrontation with a same-sex intruder (77). Accordingly, in highly aggressive hamsters, progesterone infusion in estrogen-primed females abolished aggression (33). In another study, dihydrotestosterone infusion alone or combined with estradiol increased aggression in female rats (78). Of note, in those females, estradiol alone did not affect aggressive behavior. Again, those early studies (33, 78) somehow challenge the hypothesis that the neural circuitry of aggression is completely feminized in early development in females and as a result, it becomes irresponsive to sex steroids such as testosterone (4, 76).

Complementary evidence of the participation of sex hormones in non-lactating female aggression comes from studies using chemo- and optogenetics to manipulate the activity of ERα-positive neurons and knocking down the estrogen receptors in specific brain regions. ER-α knockout female mice (ERKO) were reported to display high levels of aggression towards gonadectomized and/or estrogen-primed female intruders (79). Ovariectomy only affected aggressive behavior in those animals when performed before aggression testing, meaning that once those animals displayed aggression towards an intruder for the first time, ovariectomy did no longer affect their aggressive behavior (79). This suggests that aggressive experience overrules circulating levels of sex steroids, in line with that aggression training is reported to overshadow the effect of the estrous cycle on aggressive behavior displayed by isolated rats (23). Later studies refined this approach demonstrating that specific knockdown of ER-α within the VMH increased aggressive behavior towards juveniles in female Wistar rats (80).

Recent studies have further dissected the participation of progesterone receptor (PR) and ER-α^+^ neurons (co-expressed in a ratio of 1:1 in the VMHvl) within the VMHvl on female aggression. Briefly, ER-α-PR^+^ neurons in the medial part of the VMHvl were found to be specifically activated during aggression in virgin and lactating Swiss mice. Additionally, inhibition and stimulation of those neurons reduced and enhanced aggression in Swiss mice, respectively, regardless of the reproductive state (47). Interestingly, stimulation of Erα-PR^+^ neurons did not induce aggression but only mount behavior in C57 virgin females. However, stimulation and inhibition of those neurons in lactating C57 females resembled the effects seen in lactating Swiss females (47). Altogether, those results indicate that i) Erα^+^ neurons within the VMHvl are important for the display of aggression in females independent of the reproductive state and ii) strain differences might impact aggression in female mice.

Furthermore, another study has refined these previous findings by showing that among the ER-α^+^ neurons in the VMHvl, particularly neuropeptide Y receptor 2 (Npy2r) expressing neurons are crucial for the display of aggression in females, regardless of the reproductive state (virgin x lactating). Strikingly, stimulation of those neurons was able to induce aggression in C57 virgin females (towards adult male and female intruders) even during ongoing sexual behavior. In fact, VMHvl ERα-Npy2r^+^ (also called β cells) were suggested to suppress sexual behavior by inhibiting ER-α-Npy2r^-^ (α cells) (64). Thus, those findings i) confirm the existence of subpopulations of neurons within the VMHvl-ERα population controlling aggressive or sexual behaviors in female mice, ii) suggest that the neural circuitry of aggression is normally suppressed rather than absent in females and iii) suggest that the internal states such as lactation might inhibit sexual behavior by disinhibiting/activating aggression promoting neurons. Yet it remains to be addressed i) which are the endogenous factors leading to the shift between β and α cells activity, ii) the mechanisms by which these cells might inhibit each other (as the VMHvl ERα neurons are glutamatergic) and iii) whether the release/neurotransmission of the endogenous neuromodulators, i.e. NPY or estrogens, binding to its respective receptors resemble the effects of chemo- and optogenetic manipulations on behavior.

Finally, apart from ERα^+^ neurons, aromatase-positive neurons within the MeA amygdala were found to be necessary for maternal and intermale aggression (43). However, whether those neurons and aromatase activity also play a role in non-lactating female aggression still needs to be investigated. Of note, the expression of aromatase in fibers surrounding the VMHvl is sexually dimorphic and sensitive to the organizational effects of estrogen (76). Thus, aromatized estradiol may constitute the endogenous signal activating ERα^+^ neurons and subsequently triggering aggression in both sexes (47, 81). Additionally, the fact that aromatase fibers in the VMHvl are less abundant in females than in males (76) might explain the differences in the levels of aggression seen in mice.

### Oxytocin

Oxytocin is produced in the paraventricular (PVN) and supra-optic nucleus (SON) of the hypothalamus. Magnocellular OXT neurons release OXT into the bloodstream from the neurohypophysis during suckling and birth (82). However, within the brain, OXT release and signaling are known to be essential for the display of various social behaviors including aggression, for a review, please see (40, 82, 83). Typically in male mice and rats, OXT is reported to have anti-aggressive effects (40, 84–88). Consistent with the fact that during lactation the OXT system is up-regulated with elevated peptide content, OXT release, and OXT receptor (OXTR) binding (5, 82), maternal aggression was found to trigger OXT release in several regions within the aggression network, namely the PVN (89), CeA (89), BNST and LS (5).

In virgins, the role of the endogenous OXT system on aggressive behavior has been less studied. In the highly aggressive rodent species such as California mice, elevated levels of plasma OXT were found after an aggressive encounter, when housed in long days. This was accompanied by a higher number of OXT and pERK neurons in the PVN of long-day versus short-day housed females, independently of being exposed to aggression (90). Accordingly, high- and abnormally aggressive PWSI female Wistar Rats exhibit elevated levels of OXT mRNA in the PVN (28). Similar findings were obtained in highly aggressive IST rats with IST females showing elevated OXT release in their cerebrospinal fluid (CSF) and in the LS after and during the FIT, respectively. Importantly, those animals exhibited higher OXT release (CSF and locally in the LS) when compared to low-aggressive GH controls. Furthermore, OXT content positively correlated with aggression (23). Additionally, activation of OXT neurons in the PVN and SON *via* chemogenetics as well as stimulation of OXT axons in the vLS, by optogenetics, enhanced the mild levels of aggression displayed by GH females (23). Likewise, infusion of synthetic OXT i.c.v. or locally in the CeA exacerbated aggression in GH and IST rats, respectively (23, 27). Altogether, these data strongly indicate that OXT release promotes aggression in non-lactating females. This was further confirmed by the blockade of OXTRs either intracerebroventricularly (i.c.v.) or locally within the vLS in IST rats, OXTR antagonist (OXTR-A) was found to reduce the high levels of aggressive behavior displayed by IST females, independent of the site of infusion (23). These data strongly suggest a pro-aggressive effect of OXT in virgin females.

However, contrasting data have also been reported in rats and hamsters. In group-housed mildly aggressive female Wistar rats, exposure to the FIT decreased the activation of OXT neurons (measured by pERK) in the PVN (25). Although this might seem contradictory at first glance, one could hypothesize that this lower activity of the OXT neurons reflects the elevated release during the aggressive encounter. Therefore further studies should address how OXT neuronal activity relates to OXT release during aggression. Furthermore, synthetic infusion with OXT was found to decrease aggression in virgin LAB and NAB (non-selected for anxiety-related behavior, i.c.v.) (25) and hamsters (into the MPOA-AH) (91). Those pharmacological results should be interpreted carefully as cross-reactivity of synthetic OXT binding to vasopressin 1a receptor (V1aR) has been reported in the context of aggression (87, 92, 93). In fact, i.c.v. OXT infusion was found to decrease aggression in IST female rats, however, those effects were mediated *via* V1aRs (23).

Further evidence of the pro-aggressive role of OXT comes from the fact that OXTR binding is upregulated in lactating females in areas such as the LS, BNST, and MPOA (94). Interestingly, in highly aggressive virgin rats, OXTR binding was found to be reduced in the vLS (23) and nucleus accumbens (NAc) (28) but increased in the CeA (27). Those results although contradictory might only reflect differences in local OXT release as both increased and decreased OXTR binding have been associated with enhanced OXT release, for a review please see (82, 83). The decreased OXTR binding in the NAc is particularly interesting as escalated female aggression was shown to induce plasticity changes in this region in hamsters (32, 34).

Taken together, these data support a pro-aggressive role of endogenous OXT independently of the reproductive state. From an evolutionary point of view, adopting the same system to regulate lactational and non-lactational female aggression is to be expected. Especially, knowing that during lactation, both aggressive behavior (4, 5) and OXT system activity (increased synthesis, release, and receptor binding) are elevated (5, 82). It remains to be addressed, however, whether OXT acts on the same neuronal pathways/populations to trigger aggression in both states (virgin x lactating) and whether differences in the severity of aggressive behavior in lactating females are also reflected by changes in the OXT system (receptor binding, release, and signaling).

### Vasopressin

Vasopressin is the sister peptide of OXT. It is mainly produced in the PVN and SON, but parvocellular vasopressin expressing neurons are also found in the BNST, MeA, suprachiasmatic nucleus of the hypothalamus, and olfactory bulb (95, 96). Of note AVP positive cell bodies and axon terminal density, differ significantly across sexes, with males typically exhibiting more cell bodies in the BNST and MeA and higher fiber density in various hypothalamic regions and in the LS (95). Therefore, AVP has been suggested to drive male-typical social behaviors such as aggression (40, 83).

Accordingly, local AVP release was reported after aggression in male Wistar rats within the LS (40, 97). However, the link between AVP release/signaling and intermale aggression appears to be more complex depending on several factors such as levels of aggression (97), brain region (97), traits of anxiety (98), the display of abnormal aggression (98, 99) and dominance (100), for a review please see (40, 101).

Similar to males, the role of AVP on aggression displayed by lactating females seems to be intricate as well. Although AVP release within the CeA has been described during the display of maternal aggression in HAB rats (102), i.c.v. infusion of synthetic AVP was shown to decrease aggression in stressed lactating rats (103). Additionally, blockade of V1aRs was found to increase maternal aggression in Sprague-Dawley rats (104, 105), suggesting an anti-aggressive role of endogenous AVP. Yet, early life experiences also seem to affect the link between maternal aggression and AVP, as plasma AVP was found to be positively and negatively correlated with maternal aggression in PB stressed and control female Wistar rats, respectively (26).

In non-lactating females, compelling evidence has suggested an anti-aggressive role of the AVP system on aggression. In Syrian hamsters, infusion of synthetic AVP into the AH was shown to decrease female aggression (30). Additionally, AVP neurons in the PVN were not activated after aggression exposure in dominant female hamsters (30). In female Wistar rats, decreased or blunted AVP release centrally (CSF) or locally within the LS was reported after or during the FIT, respectively (23). Interestingly, highly aggressive IST females showed reduced AVP release in the dLS when compared to low-aggressive GH females. Accordingly, infusion of synthetic AVP either i.c.v. or within the dLS was shown to reduce female aggression in IST rats. Further evidence of the anti-aggressive effects of the endogenous AVP system on female aggression, comes from the fact that blockade of V1aRs in the dLS (region rich in V1aR but not OXTR binding) increases aggression in female Wistar rats independently of the level of aggression or housing condition of those animals (GH x IST) (23). Contrasting results have been obtained for the CeA where AVP was described to instigate aggression in highly aggressive IST females (27). Interestingly, blockade of V1aRs within the CeA did not affect aggression, indicating that the endogenous AVP release locally is not necessary for the display of female aggression (27).

Regarding V1aR densities, decreased V1aR binding in the anterior BNST, lateral hypothalamus, and dentate gyrus was associated with increased aggression in abnormally aggressive PWSI female Wistar rats. Importantly, V1aRs in the anterior BNST were differently affected by PWSI in males and females, i.e. PWSI females showed a decrease whereas males an increase in receptor binding after PWSI (28). Furthermore, reduced V1aR binding was reported in the dLS of highly aggressive IST females (23). Accordingly, knocking out V1aRs (globally) in female hamsters strongly enhanced aggression (106). Taken together, these data imply a blunted AVP signaling in various limbic and hypothalamic regions to be associated with exacerbated aggression in virgin females.

Apart from V1aRs, AVP binds also to vasopressin 1 b receptors (V1bRs) which are widespread in the rodent brain (107, 108). Expression of V1bRs (109, 110), as well as stimulation of V1bR-positive terminals projecting from the hippocampal CA2 to the dLS (111), increase aggression in male mice. Those effects seem to be sexually dimorphic as activation of V1b receptors either centrally (i.c.v.) or within the MPOA or BNST did not affect maternal aggression (112–114). To the best of our knowledge, the participation of V1bRs in non-maternal female aggression has not been investigated.

Although the anti-aggressive role of AVP seems to be evident in virgin females, the AVP neuronal population (s) contributing to those anti-aggressive effects still needs to be identified. This is especially important in the context of the sex-specific modulation of aggression by septal AVP release (23, 97). Additionally, the contribution of sex-specific distribution of AVP fibers (higher in males) and V1aRs (higher in females) to those sex-dependent effects still needs to be addressed. Future studies should dissect how endogenous AVP signaling differently contributes to instigating aggression in both sexes.

### Other neuroendocrine systems

Other neuroendocrine systems have been associated with maternal aggression in rats and mice. Of note, blockade of GnRH receptors i.c.v (45). as well as activation of prolactin and CRH 2 receptors (44) were found to reduce maternal aggression. However, the role of those peptides in virgin female aggression remains unknown. Thus we will focus on two other systems, i.e., the hypothalamic-pituitary-adrenal (HPA) axis and serotonin.

#### Stress, HPA axis and corticosterone

The HPA axis, as well as corticosterone (CORT), are known to strongly modulate aggression in male rodents (56, 101). Typically both high and low plasma CORT levels, as well as increased and decreased HPA axis activity, have been associated with aggressive behavior in males (101). Additionally, early life stress which is known to hyperstimulate (activate) the HPA axis is frequently described as a consistent model for inducing exaggerated and abnormal aggression in males [for a review please see (56, 101, 115)].

In females, the link between stress, HPA axis activity, CORT, and aggression is less clear. Nevertheless, all classical early life stress protocols used in male rodents to induce pathological aggression (56, 115), i.e. maternal separation, post-weaning social isolation, and peripubertal stress are known to increase aggression in females as well. In contrast to males, maternal separation was reported to enhance maternal aggression in mice (116). In female Wistar rats, both PB and PWSI exacerbate aggression in virgin females (26, 28), for further details please see section 3.

Notably, the role of the HPA axis and CORT in virgin female aggression is largely understudied. Confrontation with an intruder in the RIT leads to increased plasma CORT in naturally aggressive female California mice (90), suggesting that aggression triggers CORT release in females. Interestingly, aggression was found to negatively correlate with plasma CORT in female Wistar rats after the FIT (23), consistent with previous data in humans and animals associating hypo- arousal and CORT with exaggerated and abnormal aggression (56, 101, 117). Similarly, in PB female rats, plasma CORT was found to negatively correlate with non-maternal aggression, those animals also showed a reduced CORT response to an elevated platform when compared to controls (26). Intriguingly, those effects seem to be reproductive state-dependent as exposition to a male intruder and consequently display of maternal aggression increases maternal anxiety and decreases maternal care (both indicators of stress) in dams (118). However, whether those behavioral differences found in mothers are reflected by alterations in the HPA axis activity is unclear.

Altogether, those findings indicate that i) stress (especially during early life) strongly impacts aggression in females, ii) as in males, aggression appears to lead to increased CORT release in females, and iii) female aggression in non-lactating rats seems to negatively correlate with plasma CORT. Future studies should address the time course between CORT secretion and aggression display in females as well as acutely manipulate CORT levels and evaluate its effects on aggressive behavior. These studies should also address the potential reproductive state-dependent effects (virgin x lactation) on the HPA axis activity and subsequent aggressive behavior.

#### Serotonin (5-HT)

The serotoninergic system is among one of the most studied neurotransmitter systems in the context of aggression. Typically, serotonin (or agonism of its receptors) is known to be anti-aggressive in males (1, 119–121). Additionally, the elevation of 5-HT levels *via* selective serotonin reuptake inhibitors (SSRIs) has been consistently used to treat pathological aggression in humans and rodent models (122).

In females, the link between serotonin and aggression is less clear. In naturally aggressive female but not male hamsters, the acquisition of dominance and display of aggression was linked to increased activation of serotoninergic neurons within the anterior and posterior dorsal Raphe nucleus. Additionally, treatment with either fluoxetine (SSRI) or OH-DPAT (selective 5-HT1a receptor agonist) exacerbated aggression in female hamsters (30). Conversely in non-lactating female Wistar rats, SSRIs (fluoxetine and escitalopram) were found to decrease aggression (23, 24). Studies in female mice support a similar picture as tryptophan hydroxylase 2 (Tph2) knockout (Tph2KO) mice showed escalated female aggression (123).

Taken together, these data suggest the participation of the serotoninergic system in non-lactating female aggression. Yet, it remains to be determined i) which are the serotoninergic neuronal populations relevant for this behavior in females, ii) the role of different serotonin receptors on female aggression and iii) how changes in serotonin neurotransmission relate to aggressive behavior in females.

## Concluding remarks

We summarized here recent findings on the neurobiological mechanisms regulating female aggression. Importantly, in the last years, several rodent models of territorial/”rivalry”related aggression have been established in female rodents modeling different aspects of this behavior. Those protocols offer to behavioral neurobiologists a wide range of possibilities to focus either on ethological aspects (naturally aggressive species), instigations protocols (co-housing, social isolation, and/or aggression training), or on abnormal and pathological aggression (MS, PWSI, and PB). Thus, the development and use of those behavioral paradigms and/or models might allow scientists to shed light on the neural underpinnings regulating aggression in both sexes.

**Figure 1:**
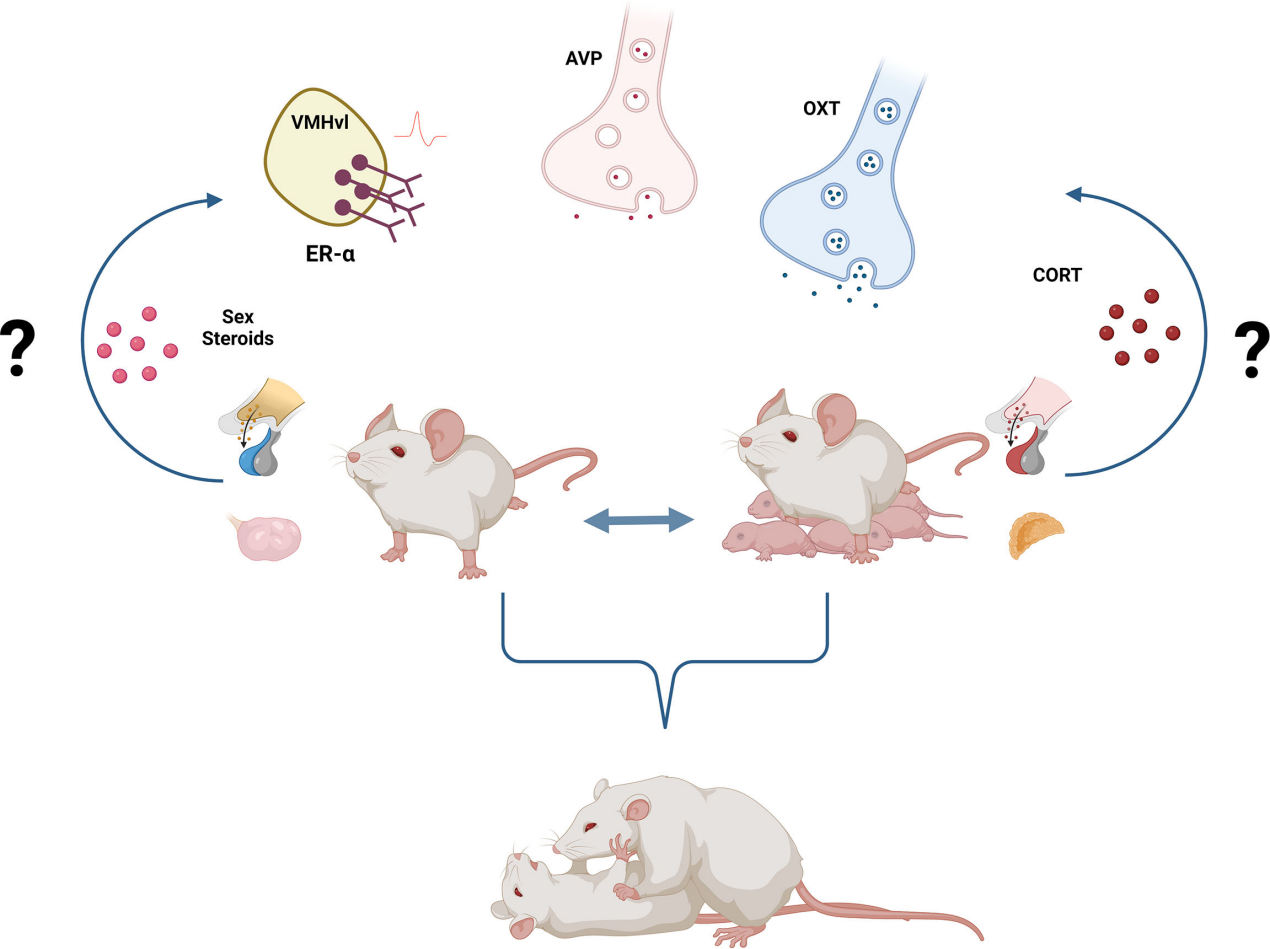
Neuroendocrine regulation of female aggression. Activation of ER-α^+^ neurons in the VMHvl as well as increased OXT and decreased AVP signaling underly female aggression, independently of the reproductive state. Yet, the activational, as well as the organizational role of sex steroids either activating ER-α+ neurons or masculinizing/feminizing the neural circuitry of female aggression remains poorly understood. Similarly, the well-established link between stress, hypothalamic-pituitary-adrenal (HPA) axis, corticosterone, and aggressive behavior needs to be further dissected in females. Created with BioRender.com. .

Regarding the neuroendocrine mechanisms underlying aggressive behavior in females, we highlighted the role of ER-α^+^ neurons within the VMHvl and of the peptides, OXT, and AVP. Interestingly, these systems were shown to regulate maternal and non-maternal female aggression similarly. As mentioned above, activation of ER-α^+^ neurons in the VMHvl, as well as OXT release, seems to promote aggression independently of the reproductive state. Additionally, AVP seems to be anti-aggressive in both lactating and virgin females. Again, co-opting the same neuroendocrine systems to regulate aggression in virgins and dams makes sense from an evolutionary point of view. However, it remains to be addressed whether i) the same neural circuits are regulating aggression in both states and ii) whether the high levels of aggression (severer attacks, for instance) in lactating females are also associated with an up-regulation of those systems, i.e. release, synthesis, receptor densities and neuronal activity (**Figure 1**).

Finally, there are still several unanswered questions regarding the neurobiological underpinnings of female aggression such as i) how does brain masculinization/feminization impacts the neural circuits regulating aggression in females (are those effects reproductive state-dependent?); ii) how does estrogen signaling affects aggression in females (are neuroestrogens necessary for female aggression?); iii) which AVP neuronal population (s) is/are relevant for female aggression?, how does AVP signaling differently impact aggression in males and females? and iv) are other neurobiological systems implicated in intermale aggression also important for female aggression?, i.e. serotonin, HPA axis, dopamine, and the reward system.

## Author contributions

VO: wrote the manuscript and drafted the figure and table with the input of JB. JB: revised the manuscript. All authors contributed to the article and approved the submitted version.

## Funding

This review was supported by the Fonds De La Recherche Scientifique - FNRS (PDR T.0015.20 to JB).

## Conflict of interest

The authors declare that the research was conducted in the absence of any commercial or financial relationships that could be construed as a potential conflict of interest.

## Publisher’s note

All claims expressed in this article are solely those of the authors and do not necessarily represent those of their affiliated organizations, or those of the publisher, the editors and the reviewers. Any product that may be evaluated in this article, or claim that may be made by its manufacturer, is not guaranteed or endorsed by the publisher.
